# Fractal wood burning remains a potentially lethal recreational activity

**DOI:** 10.1007/s12024-025-00948-2

**Published:** 2025-02-11

**Authors:** Roger W. Byard, Neil E. I. Langlois

**Affiliations:** 1 Forensic Science SA and The Adelaide School of Biomedicine, The University of Adelaide, Adelaide, South Australia Australia; 2 Adelaide School of Biomedicine, The University of Adelaide, Level 2, Room N237, Helen Mayo North, Frome Road, Adelaide, SA 5005 Australia

**Keywords:** Fractal burning, Lichtenberg burning, Wood fracking, Electrocution, Death

## Abstract

Fractal burning is a recent form of pyrography (decorative wood burning) using an extremely high voltage applied across a wood surface soaked in an electrolyte solution to produce a ferning or ‘Lichtenberg’ pattern. Two fatal cases of electrocution are reported in adults engaged in fractal burning to demonstrate characteristic features. Both had evidence of electrical contact with extensive burning. Death can occur following accidental contact with electrodes, the electrolyte solution or a loose wire, or when the victim stands on a conductive floor, or does not wear appropriate protective clothing, or has the wood on a non-insulated surface. Investigators need to check for these issues and to also determine whether the decedent had been appropriately trained in the handling of high voltage equipment.

## Introduction

Electrocution is a relatively uncommon cause of death which is most often accidental, although suicides, and more rarely, homicides do occur [1]. Fatalities result from the passage of electric current through the body causing ventricular fibrillation if the heart is involved, or respiratory paralysis if brainstem centres or respiratory muscles are affected [2]. The autopsy diagnosis of electrocution is sometimes difficult if typical electrical burns are either not obvious or have not occurred, due to a wide contact surface area such as with immersion [3].

**Fig. 1:**
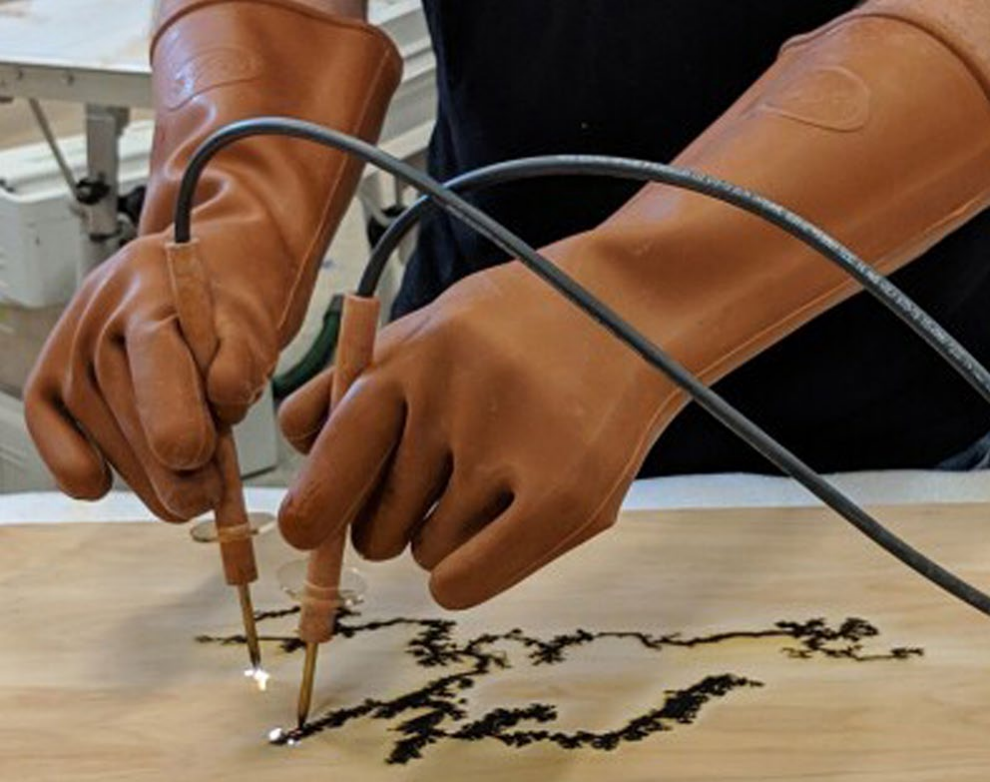
Generation of Lichtenberg burns on a wooden surface using high voltage current with appropriate safety gloves and equipment (This figure was taken from The Journal of Light Construction with permission from Nathan Rinne) [5]

An unusual form of accidental electrical death has been reported in recent years in association with fractal wood burning. This is type of pyrography (decorative wood burning) where high-voltage electric currents are used to burn branching figures into wood (Fig. 1) [4, 5]. The resultant fern pattern resembles Lichtenberg figures that are found in the skin of 17–33% of victims of lightning strike, although it may also rarely occur with high-voltage industrial contacts [6]. Despite warnings in the media and on websites about the dangers of fractal burning [5, 7] deaths and injuries continue to occur. The following two cases are reported to demonstrate the features of these activities and to illustrate certain unusual features.

## Case reports

### Case 1

**Fig. 2:**
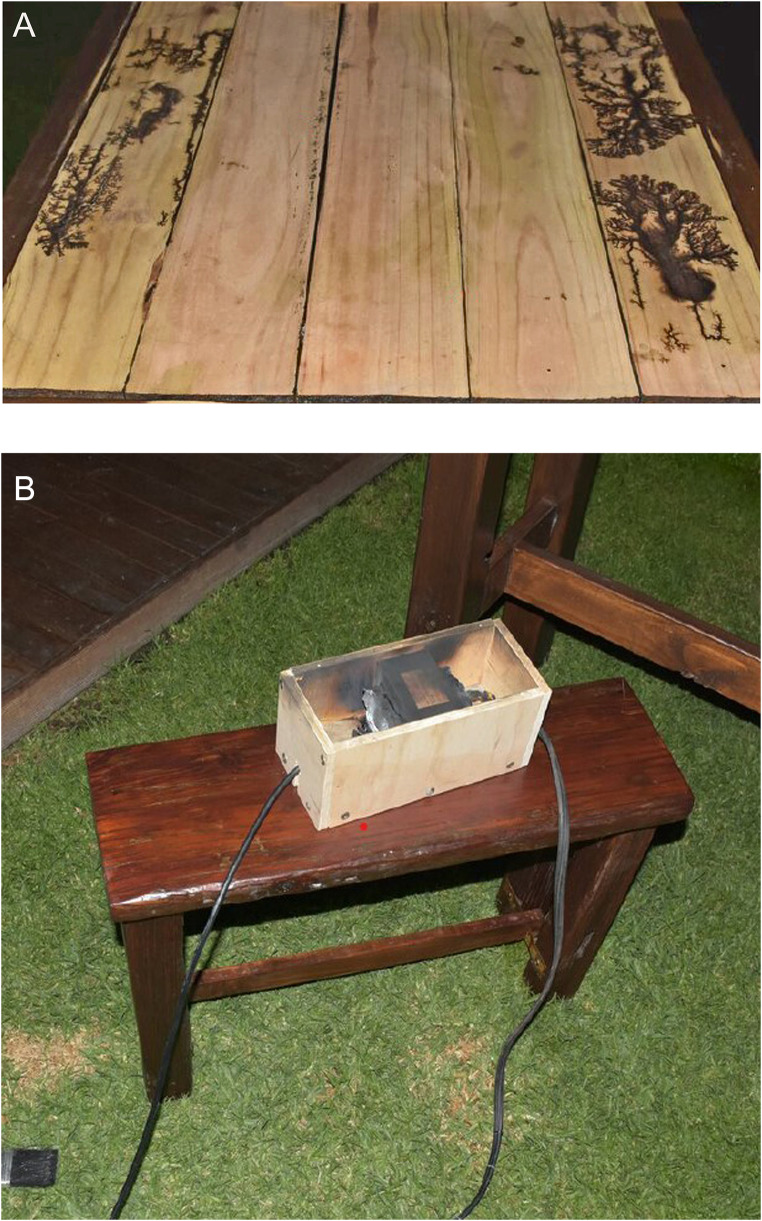
**A** Wooden planks at the scene in case 1 showing typical Lichtenberg burns. **B** A burnt out transformer near the body in case 1

An elderly female was found deceased in her backyard. She had been performing fractal wood burning using a modified transformer (Fig. 2) that had been removed from a microwave oven and connected to wires that led to metal probes. She was not wearing gloves.

**Fig. 3:**
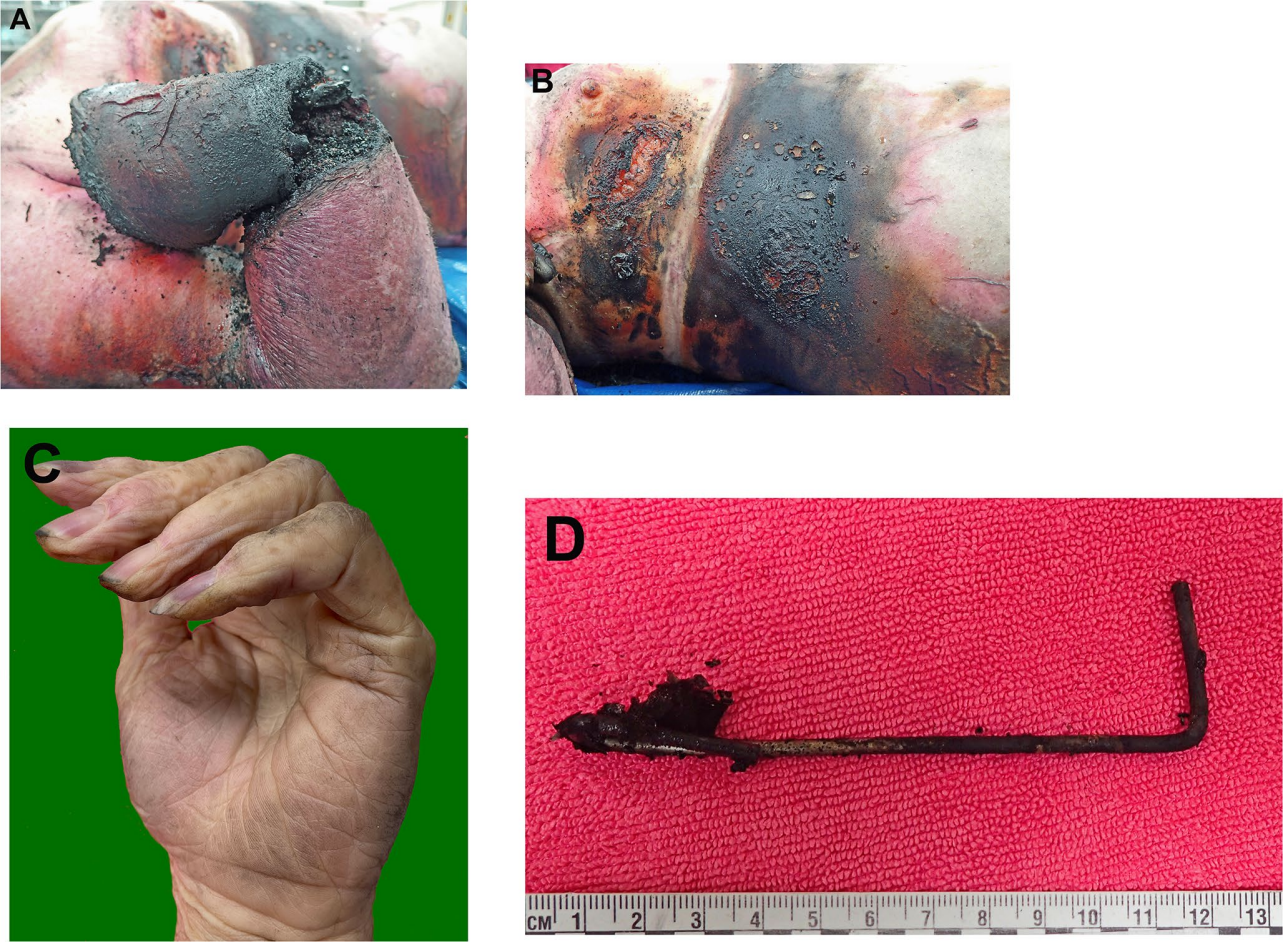
**A** Deep charring of the right hand with splitting of the skin and heat fractures of the distal radius and ulna in case 1. **B** Charring of the right side of the torso in case 1 (with a protected linear area underneath clothing). **C** The skin has split with arcing burns. Normal left hand with no electrical burns in case 1. **D** A metal electrode that was held in the right hand in case 1

On external examination with CT and toxicological analysis, she was found to have deep charring of the right hand with heat fractures of the distal radius and ulna associated with right sided burns with charring and skin splitting of the chest, abdomen and upper thigh. A metal wire was held in her right hand. Her left hand was focally soot soiled but showed no electrical burns (Fig. 3). Toxicological evaluations revealed no alcohol and only 3% carbon monoxide, with therapeutic concentrations of prescribed medications. Death was therefore attributed to electrocution associated with fractal wood burning.

### Case 2

A middle aged male was found deceased in his garage. He had been performing fractal wood burning using a transformer that he had removed from a microwave oven that was connected to two wires that were encased in wooden dowels. He was not wearing gloves.

**Fig. 4:**
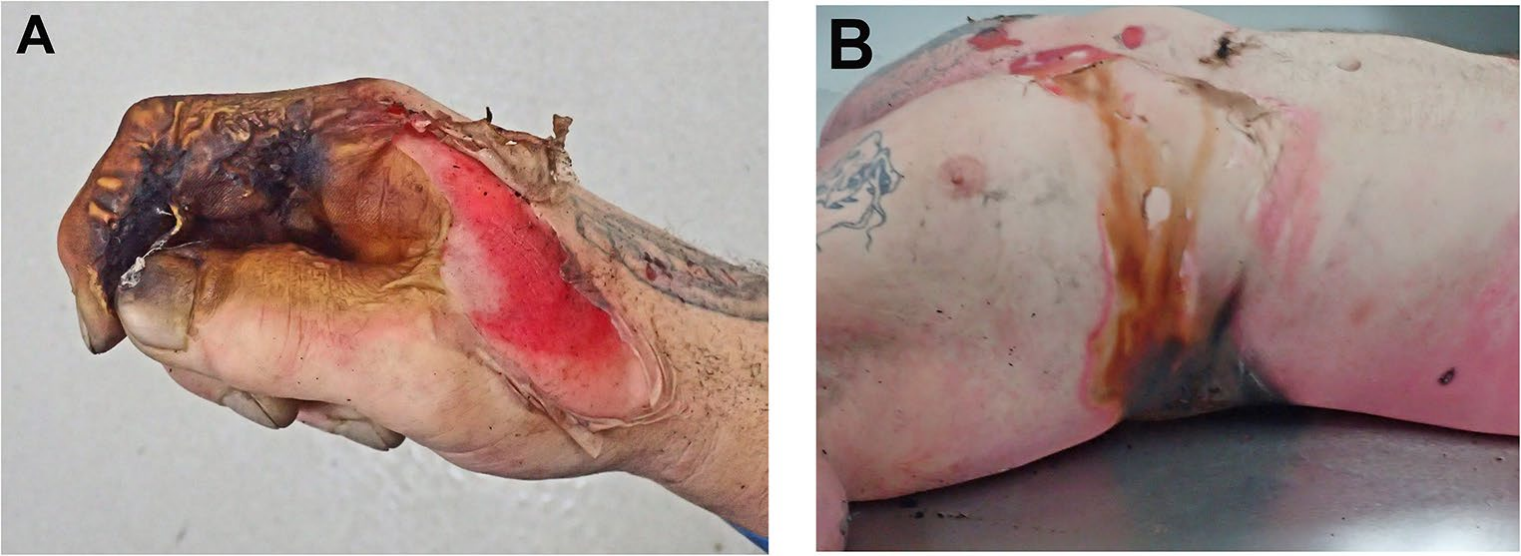
**A** Burns with charring of the right hand with blistering in case 2. **B** A typical electrical burn of the right side of the torso in case 2 with central charring, surrounding pallor and an erythematous rim

At autopsy he was found to have typical electrical burns mainly to the right hand, right arm, right side of the chest (Fig. 4), as well as to lesser degrees to the left hand and left chest. In addition, blistering was present with a ‘crocodile skin’ appearance in keeping with a high voltage electrical injury. Other findings were of superficial abrasions, lacerations and bruising of the face in keeping with a fall during the terminal episode, cardiomegaly, ischemic heart disease with previous coronary artery bypass grafting and evidence of old frontal lobe contusions. Toxicological evaluations of blood revealed 0.06% alcohol and 0.07 mg/L of methylamphetamine with no other common drugs. Death was therefore attributed to electrocution associated with fractal wood burning.

## Discussion

Fractal burning (Lichtenberg burning or wood fracking) is a recent form of pyrography or decorative wood burning where an extremely high voltage is applied between two electrodes across a wood surface that has been soaked in an electrolyte solution [4]. This results in current burning the wood with a ferning pattern. While commercial ‘Lichtenberg burners’ are available, high voltages may also be generated using home modifications of microwave oven transformers (as in both of the reported cases), with jumper leads attached to handheld electrodes [8, 9]. Other appliances that have been utilized are neon signs and oil burners [4, 8]. Microwave transformers can produce voltages ranging from 1000-15,000 with alternating currents of 0.5–2.0 amps [10]. The technique has been promoted on a number of social media sites including YouTube, TikTok and Facebook [11, 12] but may, however, result in serious injury or death [13].

The process of fractal wood burning involves dielectric breakdown whereby an insulating material actually becomes a conductor when the high voltage creates what has been termed an ‘electrical avalanche’. This is based on a rapid chain reaction of excited electrons that cause a streamer effect through break-down channels in the wood; it has been likened to an electrical detonation [8].

A problem with this activity is that individuals undertaking fractal burning have often not been trained appropriately in the handling of high voltage equipment and may not utilise basic safety precautions such as gloves and insulation. The American Association of Woodturners (AAW) has publicised these issues and has banned fractal wood burning demonstrations and equipment sales, in addition to prohibiting the display of fractal-burned art at any AAW-sponsored events and promotion of the practice through articles in AAW publications [7]. They have listed causes of electrocution that include accidental contact with an electrode, the electrolyte solution, a loose wire, or even standing on a conductive floor; in addition to the failure to wear appropriately-rated insulating protective gear, and ensuring that the wood is on an insulated surface that is not grounded. They also note that taking these precautions ‘cannot guarantee safety’ [7]. Rinne has also provided a clear list of safety steps that should be adhered to [5].

The mortality rate from inadvertent electric shock occurring during fractal wood burning is extremely high at around 71% [10], with 35 deaths recorded, the majority involving males in the United States [4, 7]. Eight cases reviewed by Herb et al. showed that all (100%) had burns to both hands suggesting that the path of the electric current was horizontally through the torso [8]. This would be in keeping with the findings in case 2. However, in case 1 it would appear that the right hand of the decedent had touched the electrode or a live part of the circuit with extreme burning of the hand, with the likely exit pathway being on the right side of the torso.

These cases demonstrate some of the issues that may arise in individuals practising fractal wood burning. The assessment of such cases requires a clear correlation of injuries documented at post mortem with the scene findings. Scene investigators must first ensure that the area is safe and that all current has been switched off and be cognisant of the standard safety instructions and materials/equipment that have been recommended for this type of potentially dangerous activity.

## Key points

1. Fractal burning is a recent type of pyrography where an extremely high voltage is applied across a wood surface.

2. Two fatal cases of electrocution are reported where modified microwave transformers were used with inadequate safety precautions.

3. Investigators need to check for evidence of contact with electrodes, the electrolyte solution or loose wires.

4. Fractal burning is promoted on social media sites, but has a high risk of death from electrocution.
